# Serum levels of bone formation and resorption markers in relation to vitamin D status in professional gymnastics and physically active men during upper and lower body high-intensity exercise

**DOI:** 10.1186/s12970-021-00430-8

**Published:** 2021-04-13

**Authors:** Jan Mieszkowski, Andrzej Kochanowicz, Elżbieta Piskorska, Bartłomiej Niespodziński, Joanna Siódmiak, Krzysztof Buśko, Blazej Stankiewicz, Dorota Olszewska-Słonina, Jędrzej Antosiewicz

**Affiliations:** 1 Department of Gymnastics and Dance, Gdansk University of Physical Education and Sport, Gdansk, Poland; 2 Department of Pathobiochemistry and Clinical Chemistry, Nicolaus Copernicus University Collegium Medicum, Bydgoszcz, Poland; 3 Kazimierz Wielki University, Department of Anatomy and Biomechanics, Institute of Physical Education, Bydgoszcz, Poland; 4 Department of Laboratory Medicine, L. Rydygier Collegium Medicum in Bydgoszcz, Nicolaus Copernicus University, Bydgoszcz, Poland; 5 Department of Bioenergetics and Physiology of Exercise, Medical University of Gdansk, Gdansk, Poland

**Keywords:** PINP, CTX, Gymnast, Vitamin D

## Abstract

**Purpose/introduction:**

To compare serum levels of bone turnover markers in athletes and non-athletes, and to evaluate the relationship between serum levels of vitamin D metabolites and exercise-induced changes in biomarker levels.

**Methods:**

Sixteen elite male artistic gymnasts (EG; 21.4 ± 0.8 years-old) and 16 physically active men (the control group, PAM; 20.9 ± 1.2 years-old) performed lower and upper body 30-s Wingate anaerobic tests (LBWT and UBWT, respectively). For biomarker analysis, blood samples were collected before, and 5 and 30 min after exercise. Samples for vitamin D levels were collected before exercise. N-terminal propeptide of type I collagen (PINP) was analysed as a marker of bone formation. C-terminal telopeptide of type I collagen (CTX) was analysed as a marker of bone resorption.

**Results:**

UBWT fitness readings were better in the EG group than in the PAM group, with no difference in LBWT readings between the groups. UBWT mean power was 8.8% higher in subjects with 25(OH)D_3_ levels over 22.50 ng/ml and in those with 24,25(OH)_2_D_3_ levels over 1.27 ng/ml. Serum CTX levels increased after both tests in the PAM group, with no change in the EG group. PINP levels did not change in either group; however, in PAM subjects with 25(OH)D_3_ levels above the median, they were higher than those in EG subjects.

**Conclusion:**

Vitamin D metabolites affect the anaerobic performance and bone turnover markers at rest and after exercise. Further, adaptation to physical activity modulates the effect of anaerobic exercise on bone metabolism markers.

## Mini-abstract

The effects of high intensity exercise on bone turnover markers in athletes and non-athletes in relation to status of vitamin D was studied. We demonstrated that adaptation to exercise and status of vitamin D modulated the response to exercise.

## Introduction

Physical exercise affects the osseous tissue by causing dynamic changes in the local mechanical conditions, thus stimulating the resident osteocytes through fluid shifts in the canalicular network [1]. Whenever a mechanical load is imposed on the osseous tissue, it induces an osteogenic response and activation of specific bone cell types (osteoclasts, osteoblasts, and osteocytes) [2]. Osteocytes effectively identify the mechanical signals, since they are located in small cavities, the lacunas, in the mineralized bone matrix. While transmitting signals to other cells, osteocytes initiate the bone tissue rebuilding process [3, 4]. The mechanism by which mechanical energy is transformed into electrical stimuli, with subsequent biochemical responses, is called mechanotransduction and plays a key role in skeletal adaptation to the actual mechanical load [5].

Typically, the human bone mass is evaluated using X-rays and by densitometry. These two methods are well known and widely used. However, they do not reflect the status of the bone as a living tissue, as they only analyse bone microarchitecture and location. Further, many scenarios call for the analysis of substances produced during bone activity and bone metabolism. Indeed, products of bone cell activity serve as markers of bone formation, and their levels reflect the bone cell function under specific circumstances, e.g. physical activity [6].

Analysis of specific biochemical markers of bone turnover may shed light on the effects of physical activity on bone metabolism during high-intensity exercise and as an effect of long-term adaptation. Further, analysis of the bone metabolic markers may inform the diagnosis and monitoring of bone metabolic disorders related to skeletal overload during highly intensive physical activity [6]. One field in which such knowledge may be applied is gymnastics, as a sport in which the bone is under pressure from specific activities, such as jumps, upheavals, and other weight-bearing activities. However, the mechanisms whereby physical exercise affects bone metabolism have not yet been fully elucidated.

Many markers of bone formation and resorption have been established. The former include bone-specific alkaline phosphatase, osteocalcin, and the C- and N-terminal propeptides of type I collagen (PICP and PINP, respectively). The latter include pyridinoline, deoxypyridinoline, and N- and C-terminal telopeptides of type I collagen (NTX and CTX, respectively) [7]. PINP and CTX have gained particular attention. PINP is released into the intracellular space and its levels reflect the number of newly formed collagen molecules [8]. On the other hand, CTX serum levels are used to determine the rate of bone turnover. CTX levels are increased during osteoporosis, osteopenia, and Paget’s disease [1, 9]. Conversely, it has been reported that lower levels of PINP are associated with hyperinsulinemia and hyperglycemia in healthy individuals [10]. Beside CTX has been reported to be a predictive factor of an increased carotid intima-media thickness in the elderly population [11]. Thus, understanding the effects of exercise on these markers can help to get inside on the mechanism of adaptation process and pro-healthy effects.

Several reports on the effect of single exercise and regular training on bone marker levels have been published; however, the data are not consistent. Without a doubt, exercise intensity and its nature, in conjunction with dietary factors, determine the bone marker response. For example, a calcium-rich meal pre-exercise reduces the levels of bone resorption markers induced by cycling [12]. Of note, many indoor athletes who practice certain sports are vitamin D-deficient [13]. Vitamin D plays an important role in bone metabolism. It also influences skeletal muscle strength, reduces muscle atrophy and parathyroid hormone concentration, and exerts many other effects [14–16]. Despite of these positive effects, to the best of our knowledge, the acute effect of exercise on bone formation and resorption markers in relation to vitamin D status had not yet been assessed. Hence, the primary aim of the current study was to evaluate the effects of vitamin D status on changes in the serum PINP and CTX levels induced by Wingate anaerobic test (WAnT) in highly trained athletes and untrained young men.

## Methods

### Ethics statement

Experimental procedures and study design were approved by the Bioethics Committee for Clinical Research at the Regional Medical Chamber in Gdansk, Poland. The study was conducted according to the 1964 Declaration of Helsinki and its later amendments. Each participant gave a written consent to participate in the study, and was informed about the purpose and test procedures, and the possibility of withdrawal of consent at any time and for any reason.

### Participants

A group of 16 elite male artistic gymnasts (EG) aged 20.6 ± 3.3 years-old and 16 physically active men (PAM) aged 19.9 ± 1.0 years-old participated in the study. Descriptive physical characteristics are shown in Table 1.

**Table 1:** Physical characteristics of the study participants (*n* = 32)

Variable	EG (*n* = 16)		PAM (n = 16)		Effect size (η^2^)
	Mean ± SD	(95% CI)	Mean ± SD	(95% CI)	
Body height (cm)	171.31 ± 3.92*	(169.21–173.40)	178.47 ± 5.08	(175.85–181.08)	0.39
Body mass (kg)	69.30 ± 6.79	(65.68–72.92)	72.99 ± 10.07	(67.81–78.17)	0.04
BMI (kg ∙ m^−2^)	23.56 ± 1.54	(22.74–24.39)	22.29 ± 3.11	(21.32–24.52)	0.01
Per cent body fat (%)	6.78 ± 3.10*	(5.12–8.44)	11.16 ± 4.83	(8.69-–3.62)	0.21

Note: *BMI* Body mass index, *EG* Elite gymnast, *PAM* Physically active man

*Significant difference between gymnasts and controls at *p* <  0.01

All gymnasts have been training regularly since 6–8 years old. (five times per week, 2-3 h per unit). At the time of the research, all gymnasts were in the highest form of preparation for participation in national and international competitions (senior level). During 1 week of training, the athletes completed from 9 to 10 training units lasting no more than 200 min (per unit). The four training session included mainly high intensity anaerobic performance. The effective duration of each session ranged from 12 to 18 min during which it was performed; jumping; squat jump; drop jump; sprint with the load; push-ups; pull up exercises including abdominal muscles, core, and arm strength training; back; and other specialized exercises using various training simulators. The remaining five training sessions included technical skills (floor exercises, pommel horse, rings, vault, parallel bars, and horizontal bar). The effective duration of a single session ranged from 40 to 50 min during which 210 to 270 gymnastic exercises were performed on selected aparatus, including 10 to 20 vault jumps. Elements from the higher difficulty groups (C, D and E) according to the Code of Points 2017–2020 International Gymnastics Federation classification constituted from 30 to 40% of all performed exercises.. PAM population declared regular participation in recreational sports, such as running, swimming, and team sports (on average, 2–3 times per week, 45–60 minunit). PAM were untrained in professional gymnastics and served as a control group for long-term gymnastic training.

All of the participants had a normal health status 3 months prior to the study, specifically, no injuries to the bone or the muscle tissue; no intake of drugs during the study; negative medical history regarding disorders of the cardiovascular system, autonomic nervous system, mental disorders, craniocerebral trauma, and other diseases that might directly affect the obtained results. The participants were informed of the nature and possible inconveniences associated with the experiment and the fact that they can opt out at any stage of the study.

### Experimental overview

The study consisted of two parts:

1. measurement of the anaerobic components of fitness using WAnT, i.e. upper and lower body high-intensity exercises;
2. assessment of the serum levels of PINP (bone formation marker) and CTX (bone resorption marker), and vitamin D metabolites in serum samples collected in part (1).

The participants were instructed to avoid caffeine, alcohol, and any substances that could have influenced their physical performance 1 month before the experiment. All participants attended a 1-h familiarization session 1 week prior to the experiment, to ensure that they were familiar with the testing equipment and procedures. Further, 48 h prior to testing, the participants were asked to refrain from exhaustive exercise, to maintain their normal dietary habits, and to come to the laboratory in a euhydrated state.

All participants performed the lower and upper body WAnT (LBWT and UBWT, respectively). The actual measurements began with LBWT. Before the test, venous blood for serum isolation was taken at rest, and 5 and 30 min after test completion. One week later, the participants completed the UBWT. Blood samples were collected at rest before and after the test, as for the LBWT.

### Measurements of the anaerobic components of fitness: LBWT and UBWT

LBWT was conducted using a cycle ergometer (Monark 894E, Peak Bike from Sweden) with MCE 5.1 software package („JBA” Zb. Staniak, Poland). For each participant, the saddle height was adjusted so that the knee remained slightly flexed after the completion of the downward stroke (with the final knee angle of approximately 170–175°). Toe clips were used to ensure that the participant’s feet were held firmly in place and in contact with the pedals. Before any experimental testing, each individual completed a standardised warm-up on the cycle ergometer (5 min at 60 rpm, 1 W/kg). Each participant was required to pedal with maximum effort for a period of 30 s against a fixed resistive load of 75 g/kg of total body mass as recommended by Bar-Or O [17].

UBWT was conducted using a hand cycle ergometer (Monark 891E). Participants sat in a chair affixed to the ground, and were advised to keep their feet flat on the ground and remain seated throughout the WAnT. The seat height and backrest were adjusted so that, with the crank position on the opposite side to the body and the hand grasping the handles, the elbow joint was almost fully extended (140–155°) and the shoulders were in line with the centre of the ergometer’s shaft. A standard resistive load equivalent to 50 g/kg of total body mass was used for each participant. Before the test, the participants completed a warm-up that involved 5 min of arm cranking at a power output of 1 W/kg and a crank rate of 60 rev/min.

For both WAnTs, each participant was instructed to cycle as fast as possible and was given a 3-s countdown before the set resistance was applied. Verbal encouragement was given to all participants to maintain their highest possible activity throughout the tests. Both cycle ergometers were connected to a PC to allow data capture via the MCE 5.1 software. The following WAnT variables were measured: peak power (W) and relative peak power (W/kg), calculated as the single highest point of power output (recorded at 0.2-s intervals); mean power (W) and relative mean power (W/kg), calculated as the average power output during the 30-s test.

### Sample collection and methodology

The blood for biochemical analyses was collected three times on the day of each test (immediately before, and 5 and 30 min after WAnT completion) into serum separation tubes (Becton Dickinson, Oxford, UK). The blood was collected at rest, fasting, and in the morning (07:00–08:00). The tubes were centrifuged at 2000×*g* for 10 min at 4 °C and stored at − 80 °C until analysis.

#### Serum CTX assay

Serum CTX levels were determined by using IDS-iSYS CTX (CrossLaps®) (Immunodiagnostic Systems, Tyne and Wear, UK), a chemiluminescence immunoassay, and iSYS analyser, according to the manufacturer’s protocol. Before the analysis, the serum aliquots were kept frozen at − 80 °C, and had only be thawed once, i.e. prior to the analysis. All analyses were performed immediately after sample thawing. Assay performance was verified by using the manufacturer-supplied controls.

#### Serum PINP assay

Serum PINP levels were determined by using IDS-[kit name] (Immunodiagnostic Systems) and iSYS analyser, according to the manufacturer’s protocol. The two-site chemiluminometric assay involved a two-point calibration in triplicate (the top calibrant value was approximately 135 μg/l), quality control material (in duplicate), and 20 μl of sample analysed in single. Specimens with PINP values above the assay range (2–230 μg/L) were diluted in specimens with low PINP levels. At each study centre, several analytical batches of specimens were analysed to establish reference intervals, by using the method during routine analyses of clinical samples. The stored specimens were analysed within 2 months of collection. Total PINP was measured electrochemiluminometrically using a Roche E170 automated analyser (Roche Diagnostics, Burgess Hill, UK, and Vivoord, Belgium). As described by the manufacturer and corroborated by published studies, the within- and between-calibration CVs of the assay were < 3.7% and < 2.9%, respectively [7].

#### Serum vitamin D assays

Vitamin D active metabolites, 25-hydroxyvitamin D_2_ [25(OH)D_2_] and 25-hydroxyvitamin D_3_ [25(OH)D_3_], as a proportion of the total serum concentration of 25-hydroxyvitamin D [25(OH)D], were analysed using the commercially available Total 25OH Vitamin D ELISA kits (BIOHIT OYJ, Helsinki, Finland), according to the manufacturer’s protocol. All assays were performed in duplicate. In the current study, the intra-assay CV for 25(OH) D was below 4%. Only samples that were not haemolysed were analysed.

### Statistical analysis

Descriptive statistics were used to analyse mean ± SD for all measured variables. The normality of distribution was checked using the Shapiro–Wilk’s test. One-way analysis of variance (ANOVA) was used to determine the difference in WAnT performance characteristics (all variables) between the EG and PAM groups. In addition, the effect size of the analysed correlations was determined (Cohen’s d-value). All calculations and graphics were generated using GraphPad Prism 6.0 (ftx.pl/program/graphpad-prism). Differences were considered statistically significant at *p* ≤ 0.05.

## Results

### Resting biochemical marker levels

No differences in the resting values of analysed biochemical markers (vitamin D metabolites, PINP, and CTX) in the EG and PAM groups were observed even though the resting procalcitonin levels in the EG group were significantly higher than those in the PAM group (Table 2).

**Table 2:** Resting values of select biochemical markers in elite gymnast (EG) and physically active man (PAM)

Variable	Unit	EG (n = 16)		PAM (*n* = 16)		Effect size (η^2^)
		Mean ± SD	(95% CI)	Mean ± SD	(95% CI)	
25(OH)D_3_	ng/ml	20.85 ± 3.93	(18.57–23.12)	23.17 ± 3.76	(21.23–25.10)	0.08
24,25(OH)_2_D_3_	ng/ml	1.38 ± 0.66	(1.00–1.77)	1.67 ± 0.84	(1.24–2.11)	0.03
25(OH)D_3_ / 24,25(OH)_2_D_3_	ng/ml	16.79 ± 4.41	(14.24–19.34)	15.40 ± 3.65	(13.52–17.27)	0.04
Procalcitonin	ng/ml	0.44 ± 0.17	(0.34–0.54)	0.27 ± 0.15*	(0.19–0.34)	0.23
C-terminal telopeptide of type I collagen	μg/l	0.52 ± 0.23	(0.37–0.63)	0.36 ± 0.28	(0.21–0.51)	0.07
N-terminal propeptide of type I collagen	μg/l	118.50 ± 43.15	(92.16–144.83)	140.05 ± 41.45	(117.05–163.04)	0.07

*Significant difference between gymnasts and controls at p <  0.01

### Anaerobic performance

**Fig. 1:**
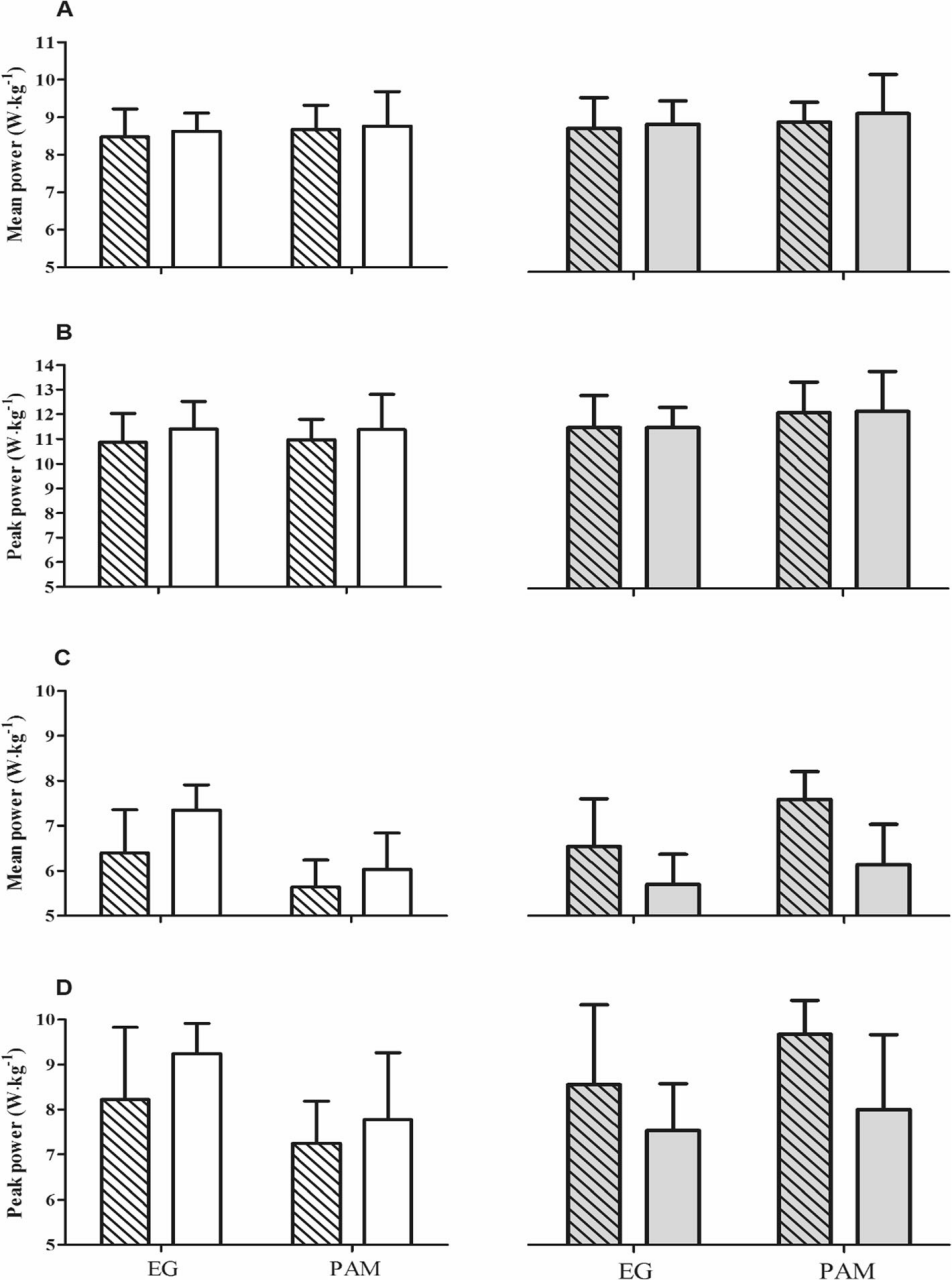
The relative mean and peak power of anaerobic exercise in elite gymnast (EG) and physically active man (PAM) depending on vitamin D3 levels. White bars with stripes, 25(OH)D_3_ levels below 22.50 ng/ml; white bars, 25(OH)D_3_ levels above 22.50 ng/ml; grey bars with stripes, 24,25(OH)_2_D_3_ levels below 1.27 ng/ml; grey bars, 24,25(OH)_2_D_3_ levels above 1.27 ng/ml; LBT, lower body test; UBT, upper body test

The LBWT and UBWT data are presented in Fig. 1 and the results of ANOVA analysis are depicted in Table 3. The analysis of variance revealed a significant effect of gymnastic training (group factor) on the mean and peak power in UBWT. Regardless of 25(OH)D_3_ and 24,25(OH)_2_D_3_ levels, these values in EG group were 14.3 and 13.3% higher than those in the PAM group. In addition, the 25(OH)D_3_ and 24,25(OH)_2_D_3_ levels significantly affected the mean power in UBWT. The values were 8.8% higher in subjects with 25(OH)D_3_ levels over 22.50 ng/ml and in those with 24,25(OH)_2_D_3_ levels over 1.27 ng/ml.

**Table 3:** Two-way (2 groups × 2 concentrations) ANOVA analysis of the body mass-normalized performance in lower body Wingate test (LBWT) and upper body Wingate test (UBWT)

Variable	Effect	df	F	p	Effect size (η^2^)	*Post-hoc* outcome
LBWT mean power	Gr	1, 28	0.40	0.53	0.01	
	D_3_ Con	1, 28	0.20	0.65	< 0.01	
	D_2_ Con	1, 28	0.64	0.64	0.02	
	Gr × D_3_ Con	1, 28	0.01	0.91	< 0.01	
	Gr × D_2_ Con	1, 28	0.05	0.82	< 0.01	
LBWT peak power	Gr	1, 28	0.01	0.94	< 0.01	
	`D_3_ Con	1, 28	1.30	0.26	0.04	
	D_2_ Con	1, 28	1.89	0.18	0.06	
	Gr × D_3_ Con	1, 28	0.02	0.87	< 0.01	
	Gr × D_2_ Con	1, 28	0.01	0.94	< 0.01	
UBWT mean power	Gr	1, 28	13.62	0.01**	0.34	EG > PAM LD_3_ < HD_3_ LD_24_ < HD_24_
	D_3_ Con	1, 28	5.63	0.02*	0.18	
	D_2_ Con	1, 28	5.70	0.02*	0.18	
	Gr × D_3_ Con	1, 28	0.94	0.33	0.03	
	Gr × D_2_ Con	1, 28	0.93	0.34	0.03	
UBWT peak power	Gr	1, 28	6.80	0.01*	0.20	EG > PAM
	D_3_ Con	1, 28	2.72	0.11	0.09	
	D_2_ Con	1, 28	2.33	0.13	0.08	
	Gr × D_3_ Con	1, 28	0.27	0.60	0.01	
	Gr × D_2_ Con	1, 28	0.42	0.53	0.01	

**Note:** *Gr* Group, *D*_*3*_ *Con* Concentration of 25(OH)D_3_, *D*_*2*_ *Con* Concentration of 24,25(OH)_2_D_3_, *LD*_*3*_ Less than 22.50 ng/ml 25(OH)D_3_, *HD*_*3*_ More than 22.50 ng/ml 25(OH)D_3_, *LD*_*24*_ Less than 1.27 ng/ml 24,25(OH)_2_D_3_, *HD*_*24*_ More than 1.27 ng/ml 24,25(OH)_2_D_3_, *EG* Elite gymnast, *PAM* Physically active man

Significant difference at *p ≤ 0.05, **p ≤ 0.01.

### Bone turnover marker levels after training

**Fig. 2:**
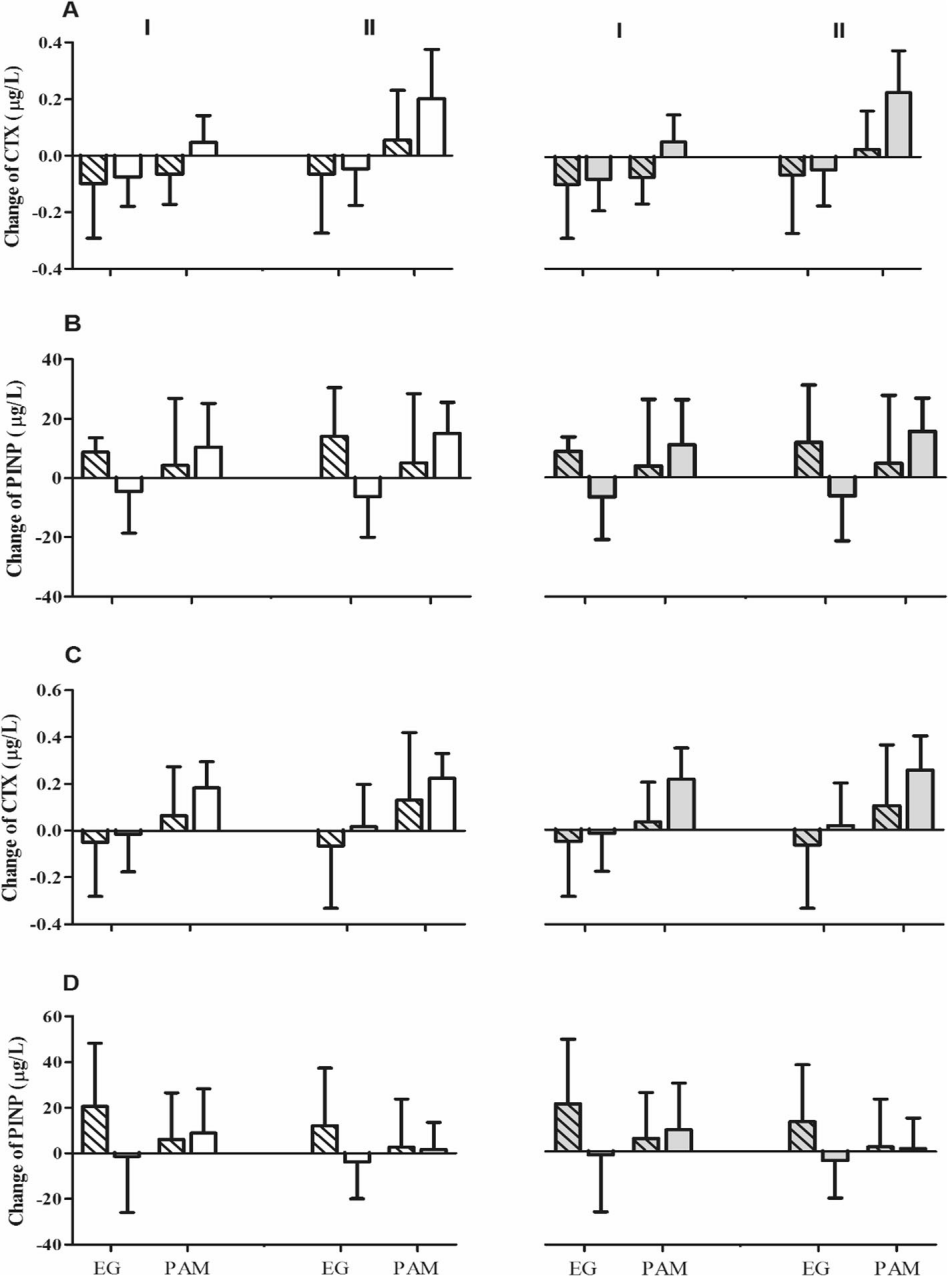
Changes in the bone turnover marker levels after Wingate anaerobic test (WAnT) in elite gymnast (EG) and physically active man (PAM) depending on the vitamin D3 levels. CTX, C-terminal telopeptide of type I collagen; PINP, N-terminal propeptide of type I collagen; **a** and **b** Lower body Wingate testing; **c** and **d** Upper body Wingate testing; I, changes in the peripheral blood 5 min post WAnT; II, changes in the peripheral blood 30 min post WAnT; white bars with stripes, 25(OH)D_3_ levels below 22.50 ng/ml; white bars, 25(OH)D_3_ levels above 22.50 ng/ml; grey bars with stripes, 24,25(OH)_2_D_3_ levels below 1.27 ng/ml; grey bars, 24,25(OH)_2_D_3_ levels above 1.27 ng/ml

Changes in the levels of bone turnover markers after LBWT and UBWT are presented in Fig. 2. The analysis of variance revealed a significant change in CTX levels 30 min post LBWT, and 5 and 30 min post UBWT (Table 4). At these time points, the CTX levels increased by 35.8, 34.2, and 49.2%, respectively, in the PAM group, but decreased by 10.9, 7.0, and 5.8%, respectively, in the EG group. Of note, the resting CTX values were significantly affected by 25(OH)D_3_ and 24,25(OH)_2_D_3_ levels. The resting CTX values were 50.2% higher in participants with 25(OH)D_3_ levels below 22.50 ng/ml and 39.5% higher in participants with 24,25(OH)_2_D_3_ below 1.27 ng/ml.

**Table 4:** Two-way (2 groups × 2 concentrations) ANOVA analysis of the bone turnover marker responses induced by lower body Wingate testing (LBWT) and upper body Wingate testing (UBWT)

Variable	Effect	df	F	p	Effect size (η^2^)	*Post-hoc* outcome
CTX rest	Gr	1, 28	2.41	0.13	0.07	LD_3_ > HD_3_
	D_3_ Con	1, 28	12.81	0.01**	0.31	
	D_2_ Con	1, 28	5.07	0.03*	0.18	LD_2_ > HD_3_
	Gr × D_3_ Con	1, 28	0.02	0.87	< 0.01	
	Gr × D_2_ Con	1, 28	0.27	0.60	0.01	
CTXchange 5 min post LBWT	Gr	1, 28	2.78	0.10	0.09	
	D_3_ Con	1, 28	2.16	0.15	0.07	
	D_2_ Con	1, 28	2.35	0.13	0.09	
	Gr × D_3_ Con	1, 28	0.93	0.34	0.03	
	Gr × D_2_ Con	1, 28	1.32	0.26	0.04	
CTX change 30 min post LBWT	Gr	1, 28	8.55	0.01**	0.24	EG < PAM
	D_3_ Con	1, 28	1.67	0.20	0.05	
	D_2_ Con	1, 28	3.04	0.09	0.11	
	Gr × D_3_ Con	1, 28	1.03	0.31	0.03	
	Gr × D_2_ Con	1, 28	2.17	0.15	0.07	
CTXchange 5 min post UBWT	Gr	1, 28	5.70	0.02*	0.18	EG < PAM
	D_3_ Con	1, 28	1.25	0.27	0,04	
	D_2_ Con	1, 28	2.72	0.11	0.09	
	Gr × D_3_ Con	1, 28	0.38	0.54	< 0,01	
	Gr × D_2_ Con	1, 28	1.27	0.26	0.04	
CTXchange 30 min post UBWT	Gr	1, 28	6.01	0.02*	0.19	EG < PAM
	D_3_ Con	1, 28	1.12	0.29	0.04	
	D_2_ Con	1, 28	2.04	0.16	0.07	
	Gr × D_3_ Con	1, 28	0.01	0.94	< 0.01	
	Gr × D_2_ Con	1, 28	0.17	0.68	< 0.01	
PINP rest	Gr	1, 28	1.85	0,22	0.07	
	D_3_ Con	1, 28	0.17	0.67	< 0.01	
	D_2_ Con	1, 28	0.22	0.63	< 0.01	
	Gr × D_3_ Con	1, 28	1.77	0.19	0.06	
	Gr × D_2_ Con	1, 28	0.33	0.56	0.01	
PINPchange 5 min post LBWT	Gr	1, 28	0.84	0.40	0.02	
	D_3_ Con	1, 28	0.30	0.58	0.01	
	D_2_ Con	1, 28	0.37	0.54	0.01	
	Gr × D_3_ Con	1, 28	2.20	0.15	0.09	
	Gr × D_2_ Con	1, 28	2.76	0.11	0.10	
PINPchange 30 min post LBWT	Gr	1, 28	0,95	0.37	0.03	EG-HD_3_ < PAM-HD_3_
	D_3_ Con	1, 28	0.58	0.45	0.02	
	D_2_ Con	1, 28	0.24	0.62	0.02	
	Gr × D_3_ Con	1, 28	4.90	0.03*	0.18	
	Gr × D_2_ Con	1, 28	3.82	0.06	0.19	
PINPchange 5 min postUBWT	Gr	1, 28	0.05	0.82	< 0.01	
	D_3_ Con	1, 28	1.09	0.31	0.04	
	D_2_ Con	1, 28	0.98	0.33	0.04	
	Gr × D_3_ Con	1, 28	1.82	0.19	0.07	
	Gr × D_2_ Con	1, 28	1.98	0.17	0.09	
PINP change 30 min post UBWT	Gr	1, 28	0.13	0.72	< 0.01	
	D_3_ Con	1, 28	1.39	0.25	0.06	
	D_2_ Con	1, 28	1.29	0.26	0.05	
	Gr × D_3_ Con	1, 28	0.78	0.38	0.03	
	Gr × D_2_ Con	1, 28	1.10	0.30	0.04	

**Note:** *CTX* C-terminal telopeptide of type I collagen, *PINP* N-terminal propeptide of type I collagen, *Gr* Group, *D*_*3*_ *Con* Concentration of 25(OH)D_2_, *D*_*2*_ *Con* Concentration of 24,25(OH)2D_3_, *LD*_*3*_ 25(OH)D_3_ levels below 22.50 ng/ml, *HD*_*3*_ 25(OH)D_3_ levels above 22.50 ng/ml, *LD*_*2*_ 24,25(OH)_2_D_3_ levels below 1.27 ng/ml, *HD*_*2*_ 24,25(OH)_2_D_3_ levels above 1.27 ng/ml, *EG* Elite gymnast, *PAM* Physically active man

Significant difference at *p ≤ 0.05, **p ≤ 0.01.

Analysis of PINP variance revealed a significant interaction of both factors only 30 min post LBWT. Further, *post-hoc* analysis indicated a 5.0% decrease in resting PINP value 30 min post LBWT in EG participants with 25(OH)D_3_ levels over 22.50 ng/ml; in PAM participants with 25(OH)D_3_ levels over 22.50 ng/ml, a 12.0% increase in resting PINP values was noted at that time point. A similar tendency was observed for participants with 24,25(OH)_2_D_3_ levels over 1.27 ng/ml; however, it was not significant (Fig. 2b).

## Discussion

In the present study, we demonstrated that WAnT induced a significantly different response in the levels of the bone resorption marker CTX in the EG and PAM groups. While CTX levels after WAnT did not change in the EG group, they significantly increased in the PAM group. This indicates that specialist gymnastic training induces adaptive changes that protect the skeletal muscle from a resorption state induced by acute exercise. Consistently, gymnasts have higher bone mineral density than runners or swimmers [18]. The effect of exercise on markers of bone formation and resorption has been evaluated in several studies; however, the findings are inconsistent [1, 12, 19]. Factors that modulate exercise-induced changes in bone marker levels remain to be identified.

### Response to WAnT of EG vs PAM

Here, we observed that the mean and peak power reached in WAnT for the arm was significantly higher in the EG group than in the PAM group. Conversely, no difference in WAnT leg performance was noted between the groups. These observations indicate that the leg power output was the same in both groups. However, the arm power output was higher in the EG group. Bearing in mind that both WAnT tests significantly increased the CTX levels only in the PAM group, it cannot be assumed that the power output, local muscle strength, or anaerobic metabolism activity are responsible for these differences.

### Response to WAnT and vitamin D

To determine whether the vitamin D status determines the effect of exercise on bone turnover marker levels, we measured the serum 25(OH) D and 24,25(OH)_2_D levels. Vitamin D greatly impacts bone metabolism; however, only limited data are available on the effect of exercise in relation to vitamin D status on bone formation and resorption markers. For instance, in professional soccer players, the resting values of bone markers were unchanged after a summer season even though a significant increase in 25(OH) D levels was observed [20]. In addition, the reports on the effect of vitamin D supplementation on marker levels are conflicting. For example, in one study involving Chines adults, vitamin D supplementation increased serum levels of bone alkaline phosphatase, but did not affect PINP and CTX levels. However, in subjects whose 25(OH) D concentration exceeded 30 ng/ml, an increase of CTX levels was observed [21]. Conversely, it has been demonstrated that improved vitamin D status in younger postmenopausal women leads to a significant reduction in serum CTX and PINP levels [22]. The findings of the current study are in agreement with the latter study, in that we observed that in both EG and PAM groups, concentration of 25(OH) D below the median value of 22.50 ng/ml was associated with higher CTX levels at baseline. Conversely, 30 min post LBWT, the increase in PINP levels was higher in the PAM group than in the EG group, but only in participants with 25(OH)D_3_ levels over 22.50 ng/ml.

It is important to note that both osteoclast and osteoblast express CYP27B1, an enzyme that converts 25(OH) D to 1,25(OH)_2_D_3_ [23]. Metabolism of 25(OH) D results in the formation of 1,25(OH)_2_D_3_, which is a critical bioactive form of vitamin D. Hence, appropriate blood levels of 25(OH) D should guarantee sufficient synthesis of 1,25(OH)_2_D_3_ by the bone cell, which can exert auto- and paracrine functions. Interestingly, concentration of the inactive form of vitamin D, 24,25(OH)_2_D_3_, below the median value of 1.27 ng/ml was also associated with higher CTX values at baseline. Another metabolic pathway, catalysed by CYP24A1, leads to formation of 24,25(OH)_2_D_3_, which has been consider to be inactive metabolite of vitamin D. However, studies of recent years indicate that it can have several biological functions. For example it can reduces the toxic effects of 1,25(OH)_2_D_3_ and is an important regulator of bone healing of fracture [24]. Besides, in individuals with renal insufficiency, treatment with 1,25(OH)_2_D_3_ leads to hypercalcemia, but this effect is reversed when the individuals are additionally treated with 24,25(OH)_2_D_3_ [25]. Our data indicates that 24,25(OH)_2_D_3_ can be an important factor regulating endocrine function of bone cells.

Collectively, the data from the current study indicate that vitamin D modulates the changes in bone remodeling marker levels in trained and untrained subjects. Further, the degree of adaptation to a specific sport and vitamin D status both impact the effect of exercise on bone metabolism markers.

### Strengths and limitations of the study

One limitation of the current study is that we only measured two metabolites of vitamin D. According to an increasing number of studies, 1,25(OH)_2_D_3_ as well as 24,25(OH)_2_D_3_, 3-epi-25(OH) D, and possibly other vitamin D metabolites play an important role in bone and calcium metabolism [24, 26, 27]. Consequently, one can expect that they also modulate the effects of exercise on bone formation and resorption. In addition, exercise by itself can modify vitamin D metabolism. For example, 30 min of cycling significantly increases serum 25(OH)D_3_ levels [19], beside endurance exercise has been shown to increase serum concentration of 24,25(OH)_2_D_3_, 3-epi-25(OH) D [28]. Further, the effect of exercise on the osteoblast and osteoclast metabolism, in addition to the direct effect of loading, is also modified by myokines, such as IL-6, IGF-1, FGF2, and others [29, 30]. Therefore, we cannot exclude the possibility that the observed modulatory effect of vitamin D on bone formation and resorption markers is also mediated by myokines.

### Ethical approval

All procedures performed in studies involving human participants were in accordance with the ethical standards of the institutional and/or national research committee and with the 1964 Declaration of Helsinki and its later amendments or comparable ethical standards.

### Informed consent

Informed consent was obtained from all individual participants included in the study.

## Data Availability

(All individual deidentified participant data are available for the next 5 years on reasonable request from the first author)
